# Prognostic Value of Long Non-Coding RNAs GUSB Pseudogene 11 in Colorectal Cancer and Its Regulatory Effect on Tumor Progression

**DOI:** 10.5152/tjg.2025.24450

**Published:** 2025-05-20

**Authors:** Jia-Rui Hu, Jie-Ting Fan, Shao-Bo Qu, Xiao-Hua He, Dai-Wei Liu, Yong-Xia Wang, Xiao-Yuan Wu, Zhan-Lin Li

**Affiliations:** TCM Oncology Department, The First Affiliated Hospital of Hebei North University, Zhangjiakou, China

**Keywords:** Colorectal cancer, GUSBP11, miR-605-3p, prognosis

## Abstract

**Background/Aims:**

Colorectal cancer (CRC) is the third most common cancer, and its progression to advanced diagnosis leads to a dismal prognosis. The long non-coding RNA (lncRNA) GUSB Pseudogene 11 (GUSBP11) can act in a variety of cancers. Nevertheless, the potential mechanism of GUSBP11 in CRC has not been reported. The purpose of this study is to investigate the relationship between the role of GUSBP11 expression in CRC progression as well as prognosis.

**Materials and Methods:**

Two hundred and fifty-nine CRC patients were recruited. Expression levels of GUSBP11 and downstream target genes in CRC cell lines were evaluated by quantitative reverse transcription polymerase chain reaction. The influence of clinical characteristics and GUSBP11 on prognosis was evaluated by the proportional hazards model. Cell-Counting-Kit-8 and transwell assays were conducted for detection of CRC cell proliferation, migration, and invasion. Dual luciferase and correlation analyses were used to validate GUSBP11 with predicted genes. Gene Ontology and Kyoto Encyclopedia of Genes and Genomes enrichment analyses were performed to analyze downstream gene function and signaling pathways.

**Results:**

The expression of GUSBP11 was upregulated in CRC and relevant to the deterioration of prognosis. The CRC cell proliferation, migration, and invasion were inhibited by GUSBP11 silencing. miR-605-3p was the downstream target gene of GUSBP11, and its expression is negatively regulated by GUSBP11.

**Conclusion:**

Taken together, this study highlights that the inhibition of miR-605-3p by GUSBP11 to regulate the downstream signaling pathway leads to prognostic malignancy and promotes tumor growth in CRC.

**Main Points**

- GUSB Pseudogene 11 (GUSBP11) is highly expressed in colorectal cancer (CRC) tissues and cells and correlates with a worse prognosis.
- The GUSBP11 was negatively correlated with the expression of the downstream target gene miR-605-3p.
- The GUSBP11 silencing upregulated miR-605-3p and inhibited the growth of CRC cell lines.
- The GUSBP11/miR-605-3p may act through parathyroid hormone synthesis and the actin cytoskeletal pathway.

## Introduction

Colorectal cancer (CRC) is the third most detected cancer and causes grave damage to public life and health. In general, the development of CRC is influenced by several factors.^1^ There is evidence that the risk of developing CRC is positively correlated with age, with a higher incidence in people aged 50 years and older.^2^ However, research data in recent years have shown that the proportion of cases in younger populations has begun to gradually increase. According to the American Cancer Society, approximately 153 020 people will develop CRC in 2023, with a mortality rate of approximately 34.34%.^3^ Of these, the incidence and mortality rates for those under 50 years of age are 12.78% and 7.14%, respectively. Although the overall data show a downward trend in CRC morbidity and mortality, the risk of CRC in people under 50 years of age is increasing, and the prognosis is not optimistic as the disease progresses toward advanced diagnosis.^3^^,^^4^ The survival rate at 5 years for patients diagnosed with CRC is nearly 90% in the early (stages I and II) and localized stages, compared to 13.1% in the advanced and metastatic stages.^2^ Therefore, it’s particularly important to explore the potential pathogenic mechanisms and therapeutic targets of CRC.

It has been found that most genomes don’t code for proteins, and these transcripts are known as non-coding RNAs (ncRNAs) which mainly consist of long non-coding RNAs (lncRNAs) and microRNAs (miRNAs).^5^ MicroRNAs are approximately 21-23 bp in length and inhibit post-transcriptional translation or downstream gene expression by particularly binding to the 3’UTR of mRNAs.^6^ The lncRNAs can be extensively involved in regulating the biological processes of cancer through different molecular mechanisms, such as regulating RNA transcription and translation, mRNA stability, and protein complex formation.^7^ Long non-coding RNAs act as competitive endogenous RNAs (ceRNAs) that compete with other genes for binding miRNAs, thereby reducing the ability of miRNAs to regulate target mRNAs.^8^ For example, highly upregulated RNAs in hepatocellular carcinoma (HCC) can serve as ceRNAs to inhibit the activity of miRNA-372 and reduce the translational repression of its target gene, *PRKACB.* ^9^ Long non-coding RNAs, as key biomarkers for the detection of cancers, play an indispensable role in the diagnostic and prognostic process of CRC. For example, a remarkable decrease in the level of lncRNA FAM30A has been detected in CRC tissues, which is related to the poor prognosis of patients.^10^

The lncRNA GUSB Pseudogene 11 (GUSBP11), which is located on human chromosome 22, has been generally studied in multiple cancers. Recent studies have shown that downregulation of GUSBP11 inhibits growth and cell metastasis of lung adenocarcinoma and promotes cell apoptosis.^11^ GUSB Pseudogene 11 is upregulated in both cervical squamous carcinoma and gastric carcinoma cell lines^12^^,^^13^ but is downregulated in triple-negative breast cell lines.^14^ The above studies suggest that GUSBP11 functions in different types of cancer cells and influences disease progression. However, the role of GUSBP11 in CRC cell lines and its prognostic value are unknown.

This study addressed the mechanism of GUSBP11’s role in CRC progression and prognosis. Based on previous studies, we hypothesized that GUSBP11 could significantly affect CRC progression. Therefore, the relationship between the roles of GUSBP11 and its downstream target genes in CRC cell lines was analyzed, and the related biological functions were investigated.

## Materials and Methods

### Patients and Samples

This study was adopted by the Ethnic Committee of The First Affiliated Hospital of Hebei North University (No. 2013-563) on November 04, 2013. All patients were informed of the purpose of the study and signed an informed consent form. The study included 259 adult CRC patients who met the following standards: (1) diagnosis with CRC, (2) age ≥ 18 years old, (3) did not undergo preoperative radiotherapy or chemotherapy, and (4) underwent surgery in The First Affiliated Hospital of Hebei North University from 2014 to 2018. Participants were followed up every 3 months for 5 years with timely updates. Adjacent normal tissue samples from those patients were taken from colon tissue at least 5.0 cm from the cancerous tissue.^15^^,^^16^

### Cell Line

The human healthy colorectal adjacent cell lines NCM-460 and CRC cell lines HCT116, HCT-15, and Caco-2 were procured from the Beijing BeNa Culture Collection (BNCC, Beijing, China). Colorectal cancer cells in the log phase were routinely subjected to digestion, and subsequent to cell resuspension at a density of 1 × 10^6^/mL, they were counted and resuspended anew. Upon reaching 80% confluence, the cells were washed with phosphate buffered solution, and the medium was replaced with a serum-free formulation. After 48 hours, the cell supernatant was collected and centrifuged at 3000 rpm/60 seconds for 10 minutes. The filtered supernatant was stored at −80°C for further use.

### Cell Transfection

HCT116 cell line was applied for the following cell function experiments because of the highest expression of GUSBP11 in it. To regulate GUSBP11 and miR-650-3p expression in vitro, the small interfering RNA sequences targeting GUSBP11 (si-GUSBP11) and its negative control (si-NC), miR-inhibitor, and inhibitor NC were designed and synthesized by BNCC (Beijing, China). HCT116 cells were cultured in 6-well plates until reaching the logarithmic growth phase, and then transfected with the corresponding sequence using Lipofectamine 2000 for 6 hours and were then placed in normal medium.

### Quantitative Reverse Transcription Polymerase Chain Reaction

Quantitative reverse transcription polymerase chain reaction (qRT-PCR) was used for mRNA detection in both tissues and cells. In brief, the total RNA from tissues and cells was isolated and extracted using the TRizol reagent, which was reverse transcribed to cDNA using the reverse transcription kit (Thermo, USA). Then, qRT-PCR was performed according to the instructions of the SYBR GREEN Quantification Kit. The system was then added to the ABI PRISM 7300 (ABI) for PCR amplification reactions. Glyceraldehyde-3-phosphate dehydrogenase or U6 served as internal controls for GUSBP11 and miR-650-3p. Relative mRNA levels were analyzed by the 2^−ΔΔCt^ method. Samples were taken in triplicate and results were acquired from 3 independent experiments.

### Cell Proliferation

For the detection of cell proliferation, a CCK-8 assay was performed. Forty-eight hours after transfection, HCT116 cells with different treatments were digested, centrifuged, suspended, and then transferred into 96-well plates at a density of 8 × 10^3^ cells/well and incubated at 37°C. Subsequently, 10 μL Cell-Counting-Kit-8 (CCK-8) reagent (Dojindo, Kumamoto, Japan) was added to each well and cultured for 4 hours at 24, 48, 72, and 96 hours, respectively. Finally, the optical density (OD) value was measured. The OD values at 450 nm were measured using a microplate analyzer.

### Cell Migration and Invasion

Cell migration and invasion were detected using a Transwell cell chamber (Corning, USA). For the cell migration assay, cells were placed in the upper chamber, and the medium containing 10% fetal bovine serum was added to the lower chambers. After incubation for 24 hours, the migrated cells were stained with 1% crystal violet for 15 minutes and were counted using a fluorescence-inverted microscope (Nikon, Tokyo, Japan). In the cell invasion assay, the majority of the steps were identical to those in the migration assay. However, it should be noted that the upper chamber was coated with Matrigel (manufactured by Becton Dickinson, Franklin Lakes, NJ, USA), which distinguished it from the migration assay.

### Bioinformatics Analysis

The downstream target miRNAs of GUSBP11 were predicted by the starBase database (https://starbase.sysu.edu.cn/). Target genes of miR-605-3p were forecasted by miRDB (http://mirdb.org/), TargetScan http://www.targetscan.org/vert_72/), and miRWalk database website. The screened overlapping genes were then highlighted by creating a Venn diagram. The function and pathway related to the screened overlapping genes were analyzed by Gene Ontology (GO) and Kyoto Encyclopedia of Genes and Genomes (KEGG) enrichment.

### Luciferase Reporter Assay

The wild type (WT) or mutant type (mut) sequence of GUSBP11 covering the miR-605-3p binding site was cloned into the pGL3 vector (Promega, Madison, WI, USA) and co-transfected into HCT116 cells with miR-605-3p mimic or mimic-NC. Forty-eight hours after transfection, luciferase activity was assayed with light protection by the Dual-Luciferase Reporter Gene Kit (E1910, Promega, Madison, WI, USA). Samples were taken in triplicate, and the results were obtained from 3 independent experiments.

### Statistical Analysis

Overall survival (OS) is the time from achieving the surgery to death. Progression-free survival (PFS) is the time from achieving the surgery to the onset of tumor progression or death. The survival curve of patients was drawn by the Kaplan–Meier (KM) plot and evaluated by the log-rank test. Proportional hazard models were also established to analyze clinical characteristics and prognostic correlations. SPSS 19.0 software (IBM SPSS Corp.; Armonk, NY, USA) was applied for data analysis. Categorical variables were compared by the *χ^2^* test, while continuous variables were analyzed via student’s *t*-test or one-way ANOVA. *P *< .05 indicates a notable difference in statistics.

## Results

### GUSB Pseudogene 11 is Highly Expressed in Colorectal Cancer Tissues and Cells

**Figure 1.**
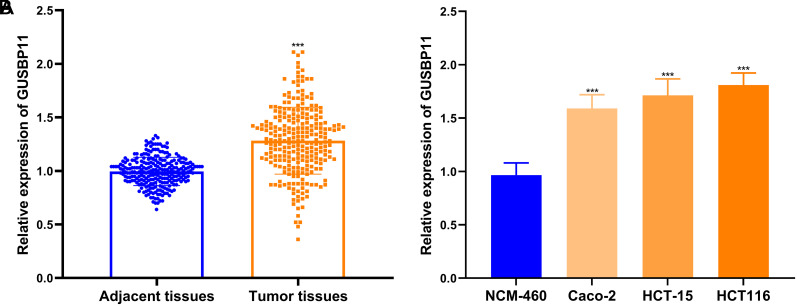
GUSBP11 was excellently expressed in CRC tissues and cells. (A) GUSBP11 was upregulated in tumor tissues. (B) GUSBP11 was upregulated in 3 CRC cell lines compared to the human nature colorectal cell line (NCM-460). *** *P* < .001.

To comprehend the potential action of GUSBP11 in CRC, its RNA levels were assessed in CRC tissues and cells along with adjacent natural tissues and cells. Compared to adjacent natural tissues, GUSBP11 expression was significantly elevated in CRC (Figure 1A). The data on the relationship between GUSBP11 and patients’ clinicopathologic characteristics showed that GUSBP11 correlated with tumor node metastasis (TNM) stage, distant metastasis, and recurrence (all *P* < .05). On the other hand, GUSBP11 was not associated with age, gender, and location (Table 1, all *P* > .05). In vitro cellular assays also showed significantly higher levels of GUSBP11 expression in HCT116, HCT-15, and Caco-2 CRC cells compared to NCM-460 (Figure 1B). Since the expression level of GUSBP11 was the highest in the HCT116 cell line compared to the other 3 CRC cell lines, HCT116 cells were selected for subsequent cellular experiments. The above data suggest that GUSBP11 is up-regulated in CRC tissues and cells, and GUSBP11 upregulation may be associated with CRC progression.

### GUSB Pseudogene 11 Expression is Related to Worsened Colorectal Cancer Prognosis

To explore the relationship between clinical characteristics and PFS, proportional hazards analysis was used. First, the univariate proportional hazards analysis indicated that GUSBP11, TNM stage, distant metastasis, and recurrence were significantly related to PFS (Table 2, all *P* < .05). Then, the above 4 significant indicators were subjected to multivariate Cox regression analysis, and it was found that all indicators could independently and significantly affect the PFS of patients (Table 2). Similarly, GUSBP11, TNM stage, distant metastasis, and recurrence were also found to be independently related to the OS of patients by univariate and multivariate Cox regression analysis (Table 3). All findings implied that GUSBP11 was likely to play an important role in influencing the PFS and survival prognosis of CRC patients, and its importance can be compared with that of TNM staging, distant metastasis, and recurrence, which is commonly used in clinical practice to assess patients’ conditions and prognoses.

**Figure 2.**
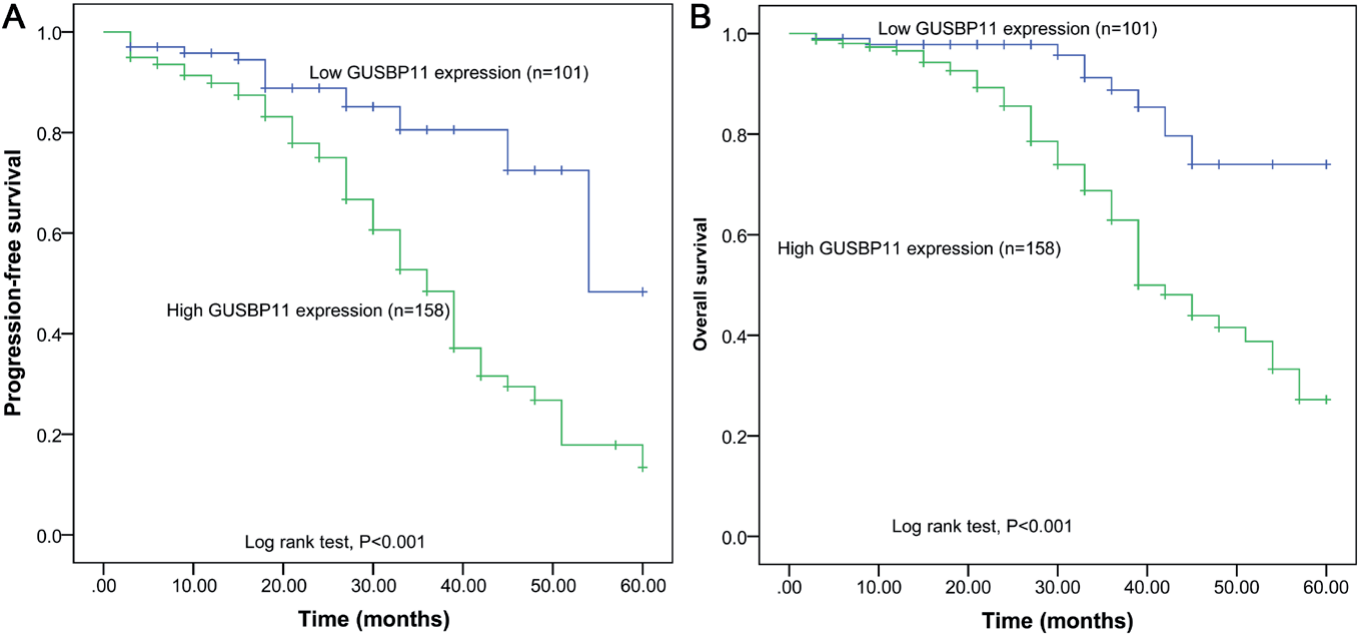
GUSBP11 expression correlates with worsened prognosis. (A) The OS of the GUSBP11 high-expression group was lower than the low-expression group and gradually decreased. (B) The PFS of the GUSBP11 high-expression group was significantly lower than that of GUSBP11 and gradually decreased. The prognosis of patients was analyzed using KM curves (PFS, OS). The log-rank test indicated a *P* < .001.

According to the mean value of GUSBP11 in tumor tissues, all CRC patients were divided into a high GUSBP11 expression group (n = 158) and a low GUSBP11 expression group (n = 101). Analysis of the survival results of the patients revealed that the OS and PFS of the GUSBP11 high-expression group were significantly shorter than those of the low-expression (Figure 2A and B), and the survival rate gradually declined over time, suggesting a worsening prognosis. The above data suggest that elevated GUSBP11 is correlated with a worsening prognosis of CRC.

### GUSB Pseudogene 11 Silencing Suppressed CRC Cell Proliferation, Migration, and Invasive Activity

**Figure 3.**
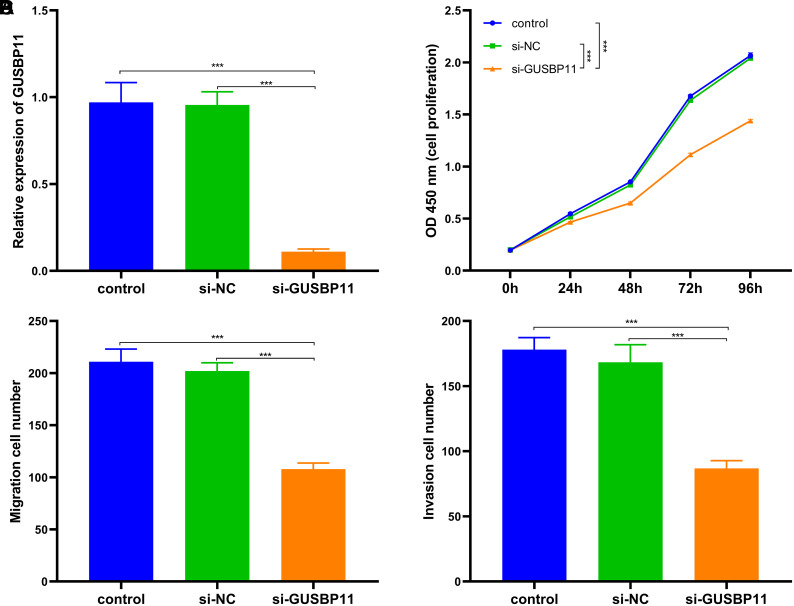
GUSBP11 silencing inhibited the growth of CRC cell lines. (A) GUSBP11 silencing inhibited GUSBP11 expression in CRC cell lines. (B) GUSBP11 silencing inhibited the proliferation of CRC cell lines. 450 nm absorbance was measured to determine the viability of CRC cell lines. (C, D) GUSBP11 silencing restrained cell migration and invasion activity. Migration and invasion of CRC cell lines after GUSBP11 silencing were analyzed by Transwell assay. *** *P* < .001.

To further validate the functional impact of GUSBP11 expression in CRC cells, *si-*GUSBP11 as well as a control group si-NC were transfected into the HCT116 cell line and confirmed the reduced expression of GUSBP11 and the success of transfection by qRT-PCR (Figure 3A). In cell proliferation assays, si-GUSBP11 transfection of HCT116 markedly restrained cell proliferation compared to the control group. (Figure 3B). Similarly, the Transwell assay revealed that the migration and cell proliferation of HCT116 were sensibly reduced after transfection with si-GUSBP11 compared to control (Figure 3C and D). The above results indicated that GUSBP11 could positively regulate the proliferation, migration, and invasion of HCT116 cells.

### GUSB Pseudogene 11 Negatively Regulates miR-605-3p Expression

**Figure 4.**
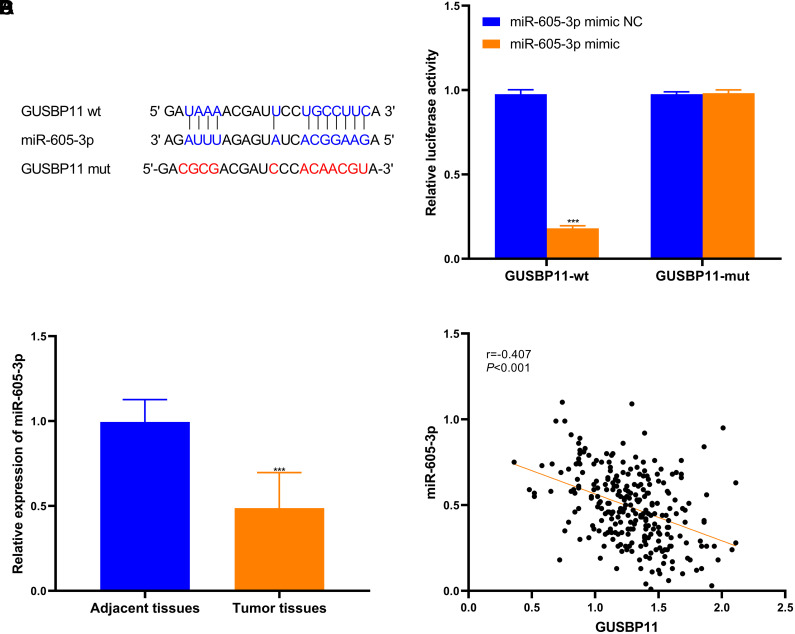
miR-605-3p interacts with GUSBP11. (A) Sequence targeting of GUSBP11 WT/mut to miR-605-3p. (B) Intracellular luciferase activity was detected after co-transfection of GUSBP11-WT/mut with miR-605-3p mimic/NC. (C) Comparison of miR-605-3p expression in adjacent tissues and tumor tissues. (D) miR-605-3p was negatively correlated with the expression of GUSBP11.

It is known that lncRNAs can bind miRNAs competitively with mRNAs to regulate downstream genes. To explore the function of GUSBP11, miRNAs that can bind to GUSBP11 were sought through starBase. Finally, miR-605-3p, which has the highest probability of targeting GUSBP11, was successfully predicted and screened, and the mutant sequence of GUSBP11 was constructed (Figure 4A). Firstly, transfection of GUSBP11-WT/mut with miR-605-3p mimics into HCT116 and assayed for luciferase activity (Figure 4B). Analysis of the results revealed that the luciferase activity was significantly depressed when GUSBP11-WT was transfected with miR-605-3p mimic, while GUSBP11-mut transfected with miR-605-3p mimic did not affect luciferase activity. The results indicated that there was an interoperability between GUSBP11 and miR-605-3p. In vitro experiments showed that miR-605-3p was eminently downregulated in CRC tissues compared with adjacent normal tissues (Figure 4C). Relevance analysis revealed that miR-605-3p was downregulated with elevated GUSBP11 expression, indicating a negative correlation between GUSBP11 and miR-605-3p (Figure 4D). In summary, miR-605-3p is a downstream target gene of GUSBP11 and is negatively regulated by GUSBP11.

### miR-605-3p Inhibits the Proliferation, Migration, and Invasive Activity of Colorectal Cancer Cells

**Figure 5.**
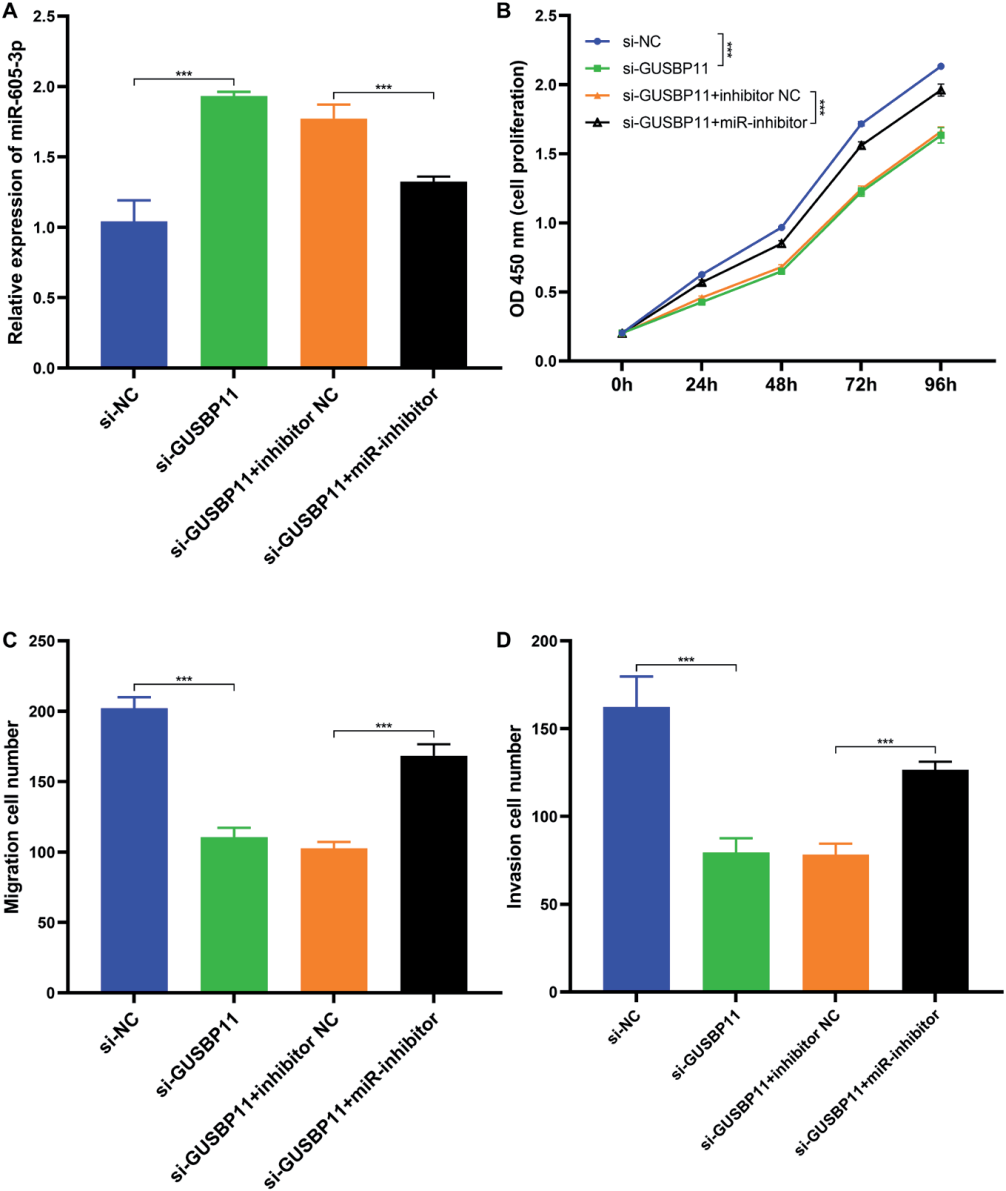
miR-605-3p upregulation restrained the growth of CRC cell lines. (A) miR-605-3p expression was upregulated in CRC cell lines after GUSBP11 silencing. (B) miR-605-3p upregulation reduced the proliferation of CRC cell lines. 450 nm absorbance was measured to determine the viability of CRC cell lines. (C, D) Upregulation of miR-605-3p inhibited migration and invasion activity of CRC cell lines. Migration and invasion of CRC cell lines after GUSBP11 silencing were analyzed by Transwell assay. *** *P* < .001.

To explore the influence of the relationship between GUSBP11 and miR-605-3p on CRC cells, it was validated by co-transfecting si-GUSBP11 and miR inhibitors into the HCT116 cell lines. After silencing *GUSBP11,* miR-605-3p expression was significantly upregulated compared to the control, whereas it was inhibited by the addition of an miRNA inhibitor (Figure 5A). Further evidence is that GUSBP11 negatively regulates the target gene miR-605-3p. Proliferation, migration, and invasion of CRC cells were also inhibited after silencing GUSBP11, which is consistent with previous results (Figure 5B-D). miR inhibitor co-transfection resulted in significantly higher proliferation, migration, and invasion of HCT116 (Figure 5B-D). The above data elucidated that miR-605-3p was restrained by GUSBP11 and negatively regulated the cell proliferation, migration, and invasion activities.

### Prediction of miR-605-3p Target Genes

**Figure 6.**
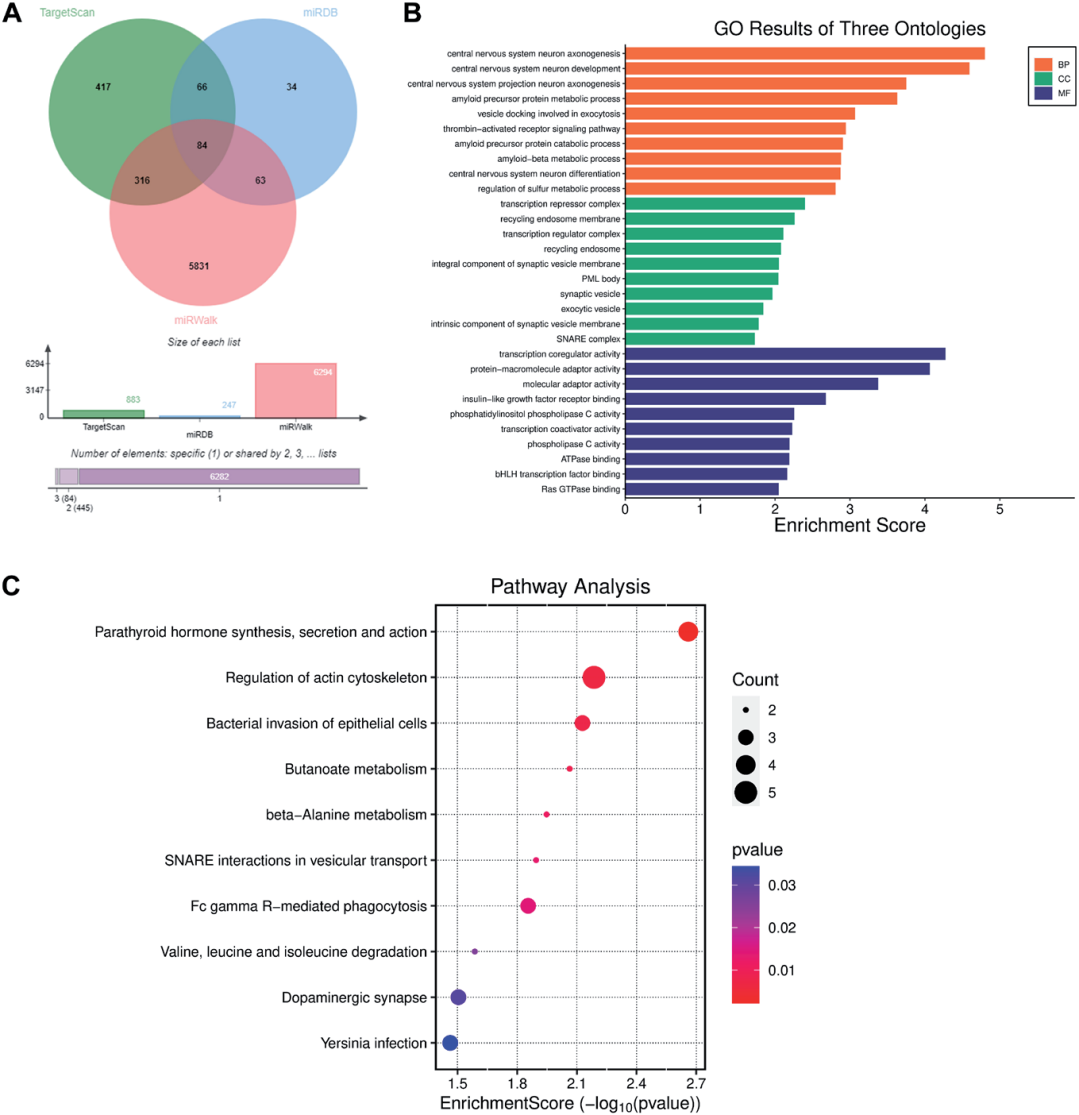
miR-605-3p target gene prediction. (A) Prediction of possible target genes of miR-605-3p by miRDB, TargetScan, and miRWalk. (B) GO enrichment analysis to calculate the function of target genes. (C) KEGG enrichment analysis to predict the signaling pathways regulated by genes.

The intrinsic mechanism by which GUSBP11 interacts with miR-605-3p to regulate downstream genes is still unknown. In this experiment, the possible target genes of miR-605-3p were screened using miRBD, TargetScan, and miRWalk. From those, 247, 883, and 6294 possible genes were respectively obtained, from which 84 overlapping genes were selected for enrichment analysis (Figure 6A). The GO analysis revealed that these genes were distributed in Biological Process, Cellular Component, and Molecular Function, which were enhanced in the neuronal developmental process, synaptic vesicle component, and transcriptase activity function respectively (Figure 6B). The KEGG analysis displayed that the above genes were mainly concentrated in the parathyroid hormone (PTH) synthesis, secretion, and actin cytoskeleton regulatory pathways (Figure 6C). The above data suggest that miR-605-3p may influence the progression of CRC by participating in PTH synthesis, secretion, and regulation of actin cytoskeleton signaling pathways.

## Discussion

Reports have demonstrated that lncRNAs are widely involved in the biological processes of cancer and that the lncRNA GUSBP11 has a potential impact on the development and prognosis of various cancers. However, its mechanism of action in CRC has not been determined. Exploring the function of GUSBP11 in CRC progression and prognosis is important for tumor regulation. In this work, GUSBP11 expression in CRC tumors and adjacent tissues was first explored. An independent cohort of tissue from CRC patients revealed that GUSBP11 was dramatically upregulated in cancerous tissues and correlated with a worsening of patient prognosis. The above discovery impelled us to further clarify the action of GUSBP11 in CRC progression.

Studies found that GUSBP11 knockdown restrains the cell proliferation and metastasis of lung adenocarcinoma and promotes apoptosis.^9^ In triple-negative breast cancer (TNBC), GUSBP11 reduces the proliferation and migration of TNBC cell lines through the regulation of miR-579-3p/SPNS2.^13^ GUSB Pseudogene 11 also inhibits the cell proliferation and migration of nasopharyngeal cancer (NPC) through the regulation of the miR-1226-3p/TM9SF4 axis to inhibit the growth of NPC cells.^14^ Therefore, the influence of GUSBP11 on tumor characteristics was searched. It was found that GUSBP11 expression was significantly correlated with TNM stage, distant metastasis, and recurrence. GUSB Pseudogene 11 is correlated with TNM staging, which implies that this gene may play a significant role in the evolution process of tumors. Higher expression of GUSBP11 may promote the activation of the proliferation signal pathways in tumor cells, leading to an increase in the volume of the primary tumor focus and thus affecting the T stage. GUSB Pseudogene 11 may also regulate the interactions between tumor cells and surrounding stromal cells as well as vascular endothelial cells, enhancing the migration and invasion abilities of tumor cells, triggering regional lymph node metastasis and distant metastasis, and thereby affecting the N stage and M stage. Further research on the changes in the expression patterns of GUSBP11 in tumor tissues at different TNM stages will help us gain a deeper understanding of the evolution process of tumors and may provide new targets and biomarkers for the development of precise treatment strategies based on TNM staging. Furtherin vitro experiments indicated that CRC cell proliferation, invasion, and migration are inhibited by GUSBP11 silencing. Interestingly, a cell growth plateau state was observed at 96 hours, and the proliferation rate of tumor cells slowed down. This may be caused by a variety of factors, such as restriction of nutrients, accumulation of metabolites, and inhibition of contact between cells. In fact, these 3 factors interact and synergistically affect the proliferation behavior of tumor cells in the cell culture system. Therefore, future studies can build a multi-factor mathematical model, comprehensively consider the interaction of these 3 factors, more accurately predict and explain the growth dynamic changes of tumor cells in the culture process, and provide a more systematic and comprehensive theoretical framework for tumor cell biology research. At the same time, the ways to break the growth plateau of tumor cells and restore their sensitivity to therapeutic drugs by regulating these factors can also be explored. Besides, more effective tumor treatment strategies can be designed based on these mechanisms, such as combination therapy based on nutrient deprivation or targeted metabolite clearance, which has important clinical translational value and application prospects.

Long non-coding RNAs negatively regulate miRNAs and suppress downstream target genes to regulate cancer progression.^17^ To further examine the underlying mechanisms of GUSBP11 in CRC, its downstream target miRNAs were explored. According to the online prediction and luciferase reporter assay, miR-605-3p was identified as a candidate target. Previously, miR-605-3p has been widely reported to be involved in the development of various cancers. For example, in HCC cells, silencing of *SNHG16* inhibited binding to and up-regulated miR-605-3p and thus inhibited HCC metastasis.^18^ Similarly, miR-605-3p can inhibit CRC progression by targeting *KIF3B*.^19^ To scrutinize the inherent mechanisms by which GUSBP11 affects CRC, the relationship of its action with its target gene, miR-605-3p, and its effect on CRC progression were explored. Consistent with previous findings, downregulated miR-605-3p was detected in CRC tissues, and it was negatively correlated with GUSBP11 expression. Furthermore, the gain and loss function experiment results determined that miR-605-3p abolished the role of GUSBP11 in CRC cell proliferation, migration, and invasive activity. It was concluded that GUSBP11 silencing suppressed CRC cell proliferation, migration and invasive activity via sponging miR-605-3p.

Numerous studies have demonstrated that miRNA dysregulation can affect cancer development by regulating cellular processes such as organ development, cell growth, and apoptosis. For example, miR-130b promotes cancer metastasis by acting as an oncomiR and downregulating TIMP-2 and the invasive activity of non-small cell lung cancer cells.^20^ Some studies have found that miR-495-3p and miR-605-3p can affect CRC progression.^19^^,^^21^ Previous studies have reported that miRNAs can bind multiple mRNAs and affect the expression of adenomatous polyposis coli by regulating the activation of downstream signaling pathways.^22^ To investigate the regulatory mechanisms by which the downstream genes of miR-605-3p affect CRC progression, a genetic screen was performed, and their possible functions and signaling pathways were predicted. Enrichment analysis is a statistical bioinformatics method for categorizing the functions of many differential genes or substances. Commonly used enrichment methods include GO and the KEGG, which analyze the functions and the signaling pathways of genes, respectively. In this study, GO analysis revealed that the downstream target genes of miR-605-3p may make a difference in neuronal developmental processes, synaptic vesicle components, and transcriptase activity functions. The KEGG results showed that the downstream target genes of miR-605-3p may function by participating in the regulation of PTH synthesis, secretion, and modulation of the actin cytoskeleton signaling pathway.

Parathyroid hormone, which is secreted by the parathyroid glands, serves as a crucial mediator in the process of bone remodeling. Literature reports that elevated levels of calcium can suppress the proliferation and differentiation of CRC cells and diminish tumor activity.^23^^,^^24^ Parathyroid hormone acts as a regulator of calcium homeostasis and modifies the translation of proteins associated with the cell cycle in CRC cells.^25^ Recent studies have indicated an increase in the serum levels of PTH among CRC patients.^26^^,^^27^ The calcium-sensing receptor, expressed in human colon epithelial cells and colon cancer cells, plays a critical role in maintaining calcium homeostasis by regulating the secretion of PTH from the parathyroid glands.^28^^,^^29^ The actin-cytoskeleton signaling pathway constitutes a key element in cell motility and has been demonstrated to be involved in the development and progression of CRC.^30^ The reorganization of the cytoskeleton is of fundamental importance for the metastatic spread of tumor cells.^31^^,^^32^ The actin-binding/binding protein Dematin has been found to interact with F-actin and activate the Rac1 signaling pathway to control cytoskeletal remodeling.^33^ Notably, DMTN is significantly downregulated in CRC tissues, and its expression level is closely correlated with advanced disease progression and a poor prognosis in CRC patients.^34^ Thus, the modulation of PTH synthesis and secretion, along with the regulation of the actin cytoskeleton signaling pathway, is likely to represent the potential underlying mechanism by which GUSBP11/miR-605-3p functions in CRC.

This study provides evidence that GUSBP11 is involved in CRC progression, identifies the downstream target gene miR-605-3p of GUSBP11, and reveals the mechanism by which GUSBP11 negatively regulates miR-605-3p to affect CRC progression and prognosis. However, the specific molecular mechanisms by which miR-605-3p regulates downstream genes and signaling pathways need to be further verified. GUSB Pseudogene 11 is promising as a therapeutic target for CRC, but because of the relatively small sample size, the actual therapeutic effects of GUSBP11 on CRC need to be investigated on a larger scale.

## Figures and Tables

**Table 1.** Clinical and Pathological Characteristics

Characteristics	Total (n = 259)	GUSBP11 Expression		*P*
		Low (n = 101)	High (n = 158)	
Age	56.753 ± 11.440	57.139 ± 11.715	56.506 ± 11.291	.665
Gender				.353
Female	124	52	72	
Male	135	49	86	
Location				.499
Colon	165	67	98	
Rectum	94	40	54	
TNM stage				.024
I + II	96	46	50	
III	163	55	108	
Distant metastasis				.008
Yes	67	17	50	
No	192	84	108	
Recurrence				.024
Yes	80	23	57	
No	179	78	101	

TNM, tumor node metastasis.

**Table 2.** Cox Regression Analysis for the Characteristics to Progression-Free Survival

Characteristics	Univariate Analysis		Multivariate Analysis	
	*P*	HR (95% CI)	*P*	HR (95% CI)
GUSBP11	.031	1.887 (1.058-3.366)	.026	1.911 (1.081-3.378)
Age	.616	1.005 (0.986-1.025)	-	-
Gender	.146	0.699 (0.432-1.132)	-	-
Location	.233	1.345 (0.826-2.191)	-	-
TNM stage	.012	1.885 (1.152-3.083)	.010	1.872 (1.162-3.015)
Distant metastasis	.000	3.690 (2.369-5.746)	.000	3.555 (2.302-5.492)
Recurrence	.000	2.721 (1.745-4.242)	.000	2.642 (1.705-4.093)

HR, hazard ratio; TNM, tumor node metastasis.

**Table 3.** Cox Regression Analysis for the Characteristics to Overall Survival

Characteristics	Univariate Analysis		Multivariate Analysis	
	*P*	HR (95% CI)	*P*	HR (95% CI)
GUSBP11	.014	2.508 (1.208-5.206)	.019	2.378 (1.155-4.894)
Age	.205	1.015 (0.992-1.039)	-	-
Gender	.477	1.216 (0.709-2.085)	-	-
Location	.899	1.038 (0.587-1.835)	-	-
TNM stage	.032	1.900 (1.058-3.413)	.015	2.040 (1.148-3.627)
Distant metastasis	.000	3.547 (2.122-5.929)	.000	3.469 (2.085-5.770)
Recurrence	.024	1.796 (1.081-2.983)	.012	1.908 (1.154-3.153)

HR, hazard ratio; TNM, tumor node metastasis.

## Data Availability

The data that support the findings of this study are available upon request from the corresponding author.
